# Metabolic biomarkers of clinical outcomes in severe mental illness (METPSY): protocol for a prospective observational study in the Hub for metabolic psychiatry

**DOI:** 10.1186/s12888-025-06579-9

**Published:** 2025-02-13

**Authors:** Arish Mudra Rakshasa-Loots, Christina Steyn, Duncan Swiffen, Katie F. M. Marwick, Robert K. Semple, Rebecca M. Reynolds, Karl Burgess, Stephen M. Lawrie, Stafford L. Lightman, Saturnino Luz, Daniel J. Smith

**Affiliations:** 1 Division of Psychiatry, Centre for Clinical Brain Sciences, University of Edinburgh, Edinburgh, UK; 2 Centre for Cardiovascular Science, University of Edinburgh, Edinburgh, UK; 3 MRC Human Genetics Unit, Institute of Genetics and Cancer, University of Edinburgh, Edinburgh, UK; 4 Institute of Quantitative Biology, Biochemistry & Biotechnology, School of Biological Sciences, University of Edinburgh, Edinburgh, UK; 5 Henry Wellcome Laboratories for Integrative Neuroscience and Endocrinology, Translational Health Sciences, Faculty of Health Sciences, University of Bristol, Bristol, UK; 6 Centre for Medical Informatics, Usher Institute, University of Edinburgh, Edinburgh, UK

**Keywords:** Metabolomics, Depression, Psychosis, Schizophrenia, Bipolar disorder, Sleep, Symptoms, Biomarkers, Circadian rhythms

## Abstract

People with severe mental illness have high rates of obesity, type 2 diabetes, and cardiovascular disease. Emerging evidence suggests that metabolic dysfunction may be causally linked to the risk of severe mental illness. However, more research is needed to identify reliable metabolic markers which may have an impact on mental health outcomes, and to determine the mechanisms behind their impact. In the METPSY research study, we will investigate the relationship between metabolic markers and clinical outcomes of severe mental illness in young adults. We will recruit 120 young adults aged 16–25 years living in Scotland with major depressive disorder, bipolar disorder, schizophrenia, or no severe mental illness (controls) for a prospective observational study. We will assess clinical symptoms at three in-person visits (baseline, 6 months, and 12 months) using the Structured Clinical Interview for DSM-5, and collect blood samples at each of these visits for agnostic profiling of metabolic biomarkers through an untargeted metabolomic screen, using the rapid hydrophilic interaction liquid chromatography ion mobility mass spectrometry method (RHIMMS). Participants will also complete remote assessments at 3 and 9 months after the baseline visit: Ecological Momentary Assessments to measure mental health, wrist actigraphy to measure rhythms of rest and activity, and continuous glucose monitoring to measure metabolic changes. Throughout the 12-month enrolment period, we will also measure objective markers of sleep using a radar sleep monitor (Somnofy). Using advanced statistical techniques and machine learning analysis, we will seek to better understand the mechanisms linking metabolic health with mental health in young adults with schizophrenia, bipolar disorder, and severe depression.

**Clinical trial number**: Not applicable

**Supplementary Information:**

The online version contains supplementary material available at 10.1186/s12888-025-06579-9.

## Introduction

People with severe mental illness have high rates of obesity, type 2 diabetes, and increased mortality from cardiovascular disease [1, 2]. These physical health inequalities are influenced by physical activity, diet and psychotropic medications, but there are also highly plausible candidate mechanisms linking metabolic dysfunction (insulin resistance, obesity, type 2 diabetes) and psychopathology (psychosis, mania, and severe depression) [3–5]. Indeed, early evidence suggests that metabolic interventions such as ketogenic diets, the insulin-sensitising drug metformin, and GLP1-receptor agonists may be effective in improving both metabolic and mental health outcomes in people with severe mental illness [6–8].

This evidence of clinical and functional response to metabolic interventions suggests an aetiological link between metabolic dysfunction and severe mental illness. However, this field of inquiry is at an early stage, and some evidence suggests that the causal link between metabolic problems and severe mental illness may be bidirectional [9]. More work is needed to identify robust metabolic biomarkers of severe mental illness across the mood-psychosis spectrum and to identify the mechanisms by which these biomarkers impact clinical outcomes. Additionally, the control of metabolism is highly circadian (with, for example, strong pulses of lipolysis overnight) and there is now considerable interest in ‘chronometabolic’ interventions (such as time restricted eating) for obesity and type 2 diabetes.

Metabolic biomarkers and psychiatric symptoms both exhibit a high degree of variation over time. Therefore, longitudinal and intensive (high-frequency) measurement is necessary to investigate associations between metabolism and mental health. For intensive measurement of mental health, short “bursts” of Ecological Momentary Assessments (EMA) can be delivered using smartphones for real-time monitoring of mental health in the context of “*life as it is lived*” [10] rather than in a clinical research setting. EMA data can therefore provide granular insights into participants’ well-being and fluctuations in their psychiatric symptoms [11, 12]. In addition to self-reported measures of well-being, objective data on aspects of well-being such as sleep quality, sleep duration, rest, and activity can be collected non-invasively using radar sleep monitors and wrist-worn actigraphs. One key index of metabolic health can similarly be measured intensively using commercially available transdermal continuous glucose monitors. These data collection methods are unintrusive, can be implemented in the participants’ everyday settings, provide dynamic data of changes across the day and night, and may be applied for longer periods without significant burden on participants.

In the METPSY research study, we will investigate the associations between metabolic biomarkers (measured using an innovative “deep phenotyping” approach) and clinical outcomes of severe mental illness in young adults. We will also investigate sleep/circadian rhythms at high resolution to assess how circadian disruption influences observed associations between metabolism and clinical outcomes. For the purposes of this protocol, “severe mental illness” refers to severe major depressive disorder (MDD), bipolar disorder, or schizophrenia. The METPSY study forms part of the Hub for Metabolic Psychiatry (www.metabolicpsychiatryhub.com), a multidisciplinary programme of work within the wider UKRI Mental Health Platform (www.mentalhealthplatform.ac.uk).

### Study objectives

The primary objective is to determine whether changes in metabolic biomarkers in young adults with severe mental illness are associated with clinical outcomes over 12 months.

Secondary objectives for this study are to:

- Explore the relationship between mental health and metabolic biomarkers in a ‘real world’ setting using burst Ecological Momentary Assessments (EMA) and Continuous Glucose Monitoring (CGM).
- Explore the relationship between metabolic biomarkers and sleep quality or rhythms of rest/activity using sleep monitors and wrist actigraphs.

## Methods

This is a prospective, observational study being carried out in Edinburgh, UK. The study design and methods have been reviewed and informed by members of a Lived Experience Advisory Group (LEAP) for the wider Hub for Metabolic Psychiatry, comprising individuals with relevant lived experience of severe mental illness. LEAP members will continue to be consulted at all stages of the study.

### Ethical approval

This study will be carried out in accordance with internationally recognised standards for ethical research and the Declaration of Helsinki. All participants will provide written informed consent to take part in the study. This study has received ethical approval from the NHS North of Scotland Research Ethics Committee (1) (REC reference: 24/NS/0138).

### Study timeline

**Fig. 1:**
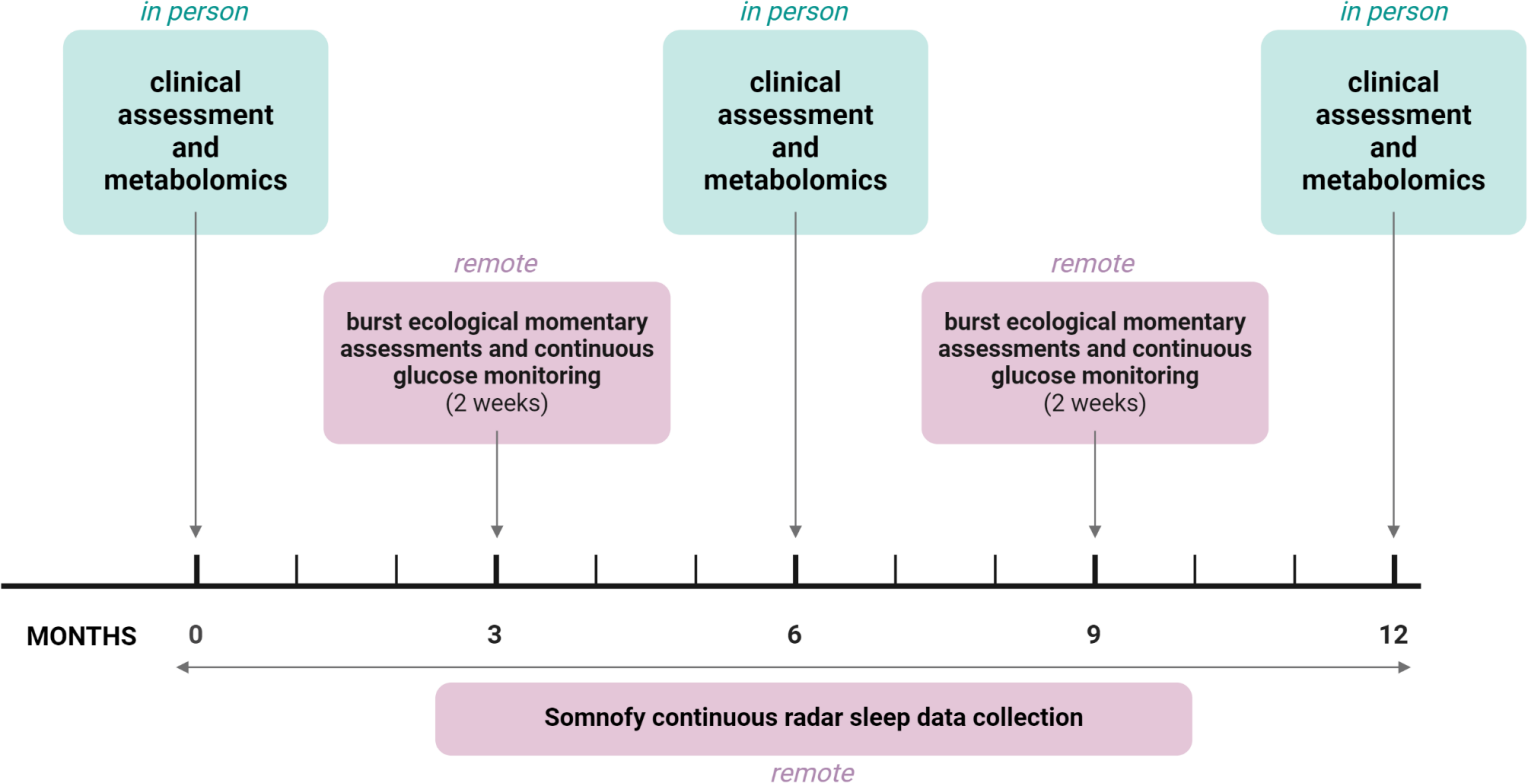
Timeline of participation and data collection procedures. Figure created with BioRender

Each participant will be enrolled in the study for 12 months in total, involving one in-person baseline assessment, two in-person prospective assessments (at 6 and 12 months), two remote “burst” assessments each lasting two weeks (at 3 and 9 months), and continuous remote sleep data collection throughout the 12 months (Fig. 1).

### Study population

This study will involve young adults (aged 16–25) with and without severe mental illness. We will focus on young adults in this study to minimise the effect of co-morbid cardiometabolic disorders

#### Sample size

We will recruit a total of *N* = 120 young adults for this study, split evenly across four groups: participants with MDD, bipolar disorder, schizophrenia, and no history of mental illness. For comparison of participants with MDD and control participants using EMA data, assuming a linear mixed model (LMM) and a conservative completion rate of 75%, *N* = 120 participants will allow the detection of medium effect sizes (*d* = 0.5) with > 80% power

#### Inclusion and exclusion criteria

Principal inclusion criteria for all participants in this study will be: age between 16 and 25 years; capacity to provide informed consent and undertake study procedures; living in Scotland; and having access to their own smartphone and a reliable internet connection at home. Participants with severe mental illness will be included if they have a diagnosis of MDD, bipolar disorder or schizophrenia, as confirmed using the Structured Clinical Interview for DSM-5 (SCID-5), and have been clinically stable for 3 months prior to enrolment in the study

Principal exclusion criteria will be: inability to complete study procedures (e.g. due to lack of proficiency in English or needle phobia); inability to use or wear devices required for data collection; or drug or alcohol dependency identified using the SCID-5

#### Recruitment pathways

Participants will be recruited through five pathways: the National Research Scotland (NRS) Mental Health Network; the National Research Scotland (NRS) Primary Care Network; the Scottish Health Research Register (SHARE); physical advertisements in relevant locations such as psychiatry clinics; and community organisations advertising the study directly to their membership in newsletters and on their social media channels. Of these pathways, SHARE, a database of volunteers who consent to the access and use of their electronic health records to identify them for participation in research, will be the primary route for identification and recruitment of participants without any severe mental illness

Equity, diversity, and inclusion is a key priority for the research team. Advertising through relevant community organisations in Scotland will enable us to actively publicise the study amongst population groups that are historically and currently underrepresented in clinical research: young women, ethnic minorities, and LGBTQ + individuals. The study leaflet for these community organisations also includes a statement encouraging people from minoritised social identities to take part in the study. Through these actively inclusive recruitment practices, we will seek to ensure that our participant sample is broadly representative of the population of people living with severe mental health issues in Scotland

### Study procedures

#### Baseline study visit

During an in-person baseline study visit, participants will provide personal data (including age, sex and gender, and ethnicity) and contact details. Using questionnaires, we will measure demographic characteristics (educational attainment, employment status, Scottish Index of Multiple Deprivation), lifestyle characteristics (smoking status, Alcohol Use Disorders Identification Test [AUDIT]), and medical history (current medication use, including hormonal medication, and recent infection history). Participants will undergo anthropometry (blood pressure, height, weight, waist-to-hip ratio, mid-arm circumference, bio-impedance) and a comprehensive psychiatric assessment involving the SCID-5 and the nine-item Patient Health Questionnaire (PHQ-9). At this baseline study visit, participants will also receive a radar sleep monitor, which will be used to monitor their sleep quality remotely throughout their participation in the study

Participants who are found to be eligible for the study will also provide two blood samples for biomarker analysis during this visit. Participants will be asked to refrain from eating anything and drinking anything other than water for a minimum of 3 h before their in-person study visits. Timing and details of participants’ latest meal before their in-person visits will also be recorded. Timing of blood sample collection will be harmonised between participants and documented for each sample

#### Prospective study visits

At 6 and 12 months after the baseline study visit, participants will complete in-person prospective assessments. At these prospective visits, participants will again undergo psychiatric assessment using the SCID-5 and the PHQ-9 to determine whether they experienced any relapse of psychiatric symptoms in the preceding 6 months, and provide two blood samples (collected after a minimum of 3 h of fasting) for biomarker analysis

#### Burst assessments

At 3 and 9 months after the baseline study visit, participants will complete a set of remote “burst” assessments lasting a total of 14 days each. During these burst assessments, participants will undergo EMA on their personal smartphones to identify daily changes in eating habits, menstruation, mental health, and subjective sleep quality. EMA will be delivered up to 2 times a day, at quasi-random times between 10 am and 10 pm each day, with each burst involving questions adapted from the four-item Patient Health Questionnaire (PHQ-4) [13], the Altman self-rating mania scale (ASRM) [14], the Brief Psychiatric Rating Scale [15], and the Brief Pittsburgh Sleep Quality Index (Brief-PSQI) [16]. Adaptations were made to these validated scales to make them suitable for EMA; for instance, we ask participants about their mood *right now* instead of *in the past two weeks* (PHQ-4). The adapted forms of these scales are available in Supplementary Materials. In addition to responding to EMA prompts, participants will also wear a wrist actigraph and a continuous glucose monitor throughout the 14-day burst assessment periods.

### Metabolic profiling

Blood samples will be collected in lithium-heparin collection tubes at in-person study visits, placed immediately on ice, and centrifuged to extract plasma, which will be aliquoted and stored at − 80 °C. Plasma will subsequently be analysed to quantify metabolic biomarkers using the rapid hydrophilic interaction liquid chromatography ion mobility mass spectrometry methods (RHIMMS) [17]. Metabolomic analysis will initially be untargeted, annotating and determining relative quantities of several hundred metabolites, followed by hypothesis-driven targeted approaches focusing on metabolic pathways of interest a priori, including mitochondrial function, insulin signalling, glycolysis, and the tricarboxylic acid cycle. Additional plasma and whole blood samples collected in EDTA tubes will also be stored and shared with the wider UKRI Mental Health Platform network for analysis of a range of biomarkers in future, encompassing metabolomics, proteomics, and genomics.

### Data analysis and sharing

#### Planned analyses

This study will involve different levels of analysis and modelling approaches adapted to the types of data being collected. Analysis of EMA (time-series) data in relation to clinical outcomes will employ multilevel (mixed) modelling to account for the dependencies in repeated observations per time period and subject. To investigate effects across EMA variables and passively monitored variables (such as glucose levels, rest/activity levels and sleep) we will employ the Dumitrescu-Hurlin panel Granger causality analysis [18]. We will also employ long short-term memory (LSTM) machine learning models to investigate possible non-linearity and longer-term dependencies in these time series [19]. Finally, to investigate the relationships between the cross-sectional clinical and metabolomic assessment and the EMA, continuous glucose monitoring, actigraphy, and sleep data, we will use dynamic Structural Equation Modelling approaches based on stochastic differential equations, which can account for unequally spaced measurements and heterogeneous measurement intervals [20].

#### Data sharing

A key priority for the research Hubs in the UKRI Mental Health Platform is to develop a harmonised, high-value, and widely accessible dataset for the mental health research community. Therefore, de-identified data from this study will be made available for download in line with open access principles in an established open access repository. De-identified data will also be shared within the wider UKRI Mental Health Platform collaboration, coordinated by DATAMIND (www.datamind.org.uk) through a Trusted Research Environment, so that it can be harmonised and pooled with data from the other research Hubs and used for further research.

## Conclusion

The METPSY study aims to deliver significant advances in the understanding of the mechanisms linking metabolic health and mental health. Key anticipated challenges to successful delivery of this study include attrition over time, since young adults aged 16–25 may be likely to move away for university or work, and potentially low completion rate for remote burst assessments. To mitigate these risks, this study involves a mix of active and passive data collection tools, thus representing an approach to balancing the collection of multi-modal longitudinal data in participants with severe mental illness while minimising participant burden. Principles of open science, lived experience engagement, and equity and inclusion have been carefully embedded in this research study to maximise the impact and value of this work to mental health researchers and patient communities.

## Electronic supplementary material

Below is the link to the electronic supplementary material.

Supplementary Material 1

## Data Availability

No datasets were generated or analysed during the current study.

## Abbreviations

ASRM: Altman self-rating mania scale
AUDIT: Alcohol use disorders identification test
CGM: Continuous glucose monitoring
EDTA: Ethylenediaminetetraacetic acid
EMA: Ecological momentary assessments
LEAP: Lived experience advisory panel
LSTM: Long short-term memory
MDD: Major depressive disorder
NRS: National research scotland
PHQ: Patient health questionnaire
PSQI: Pittsburgh sleep quality index
RHIMMS: Rapid hydrophilic interaction liquid chromatography ion mobility mass spectrometry method
SCID-5: Structured clinical interview for DSM-5
SHARE: Scottish health research register
