# Supplementary material for: Metabolic biomarkers of clinical outcomes in severe mental illness (METPSY): protocol for a prospective observational study in the Hub for metabolic psychiatry
Source: BMC Psychiatry. 2025 Feb 13;25:122. doi: 10.1186/s12888-025-06579-9 (PMC11827341; doi:10.1186/s12888-025-06579-9)
Supplement: Supplementary file 1 — Supplementary Material 1 [file 12888_2025_6579_MOESM1_ESM.docx]

**Ecological Momentary Assessments: Questionnaires**

***Mealtimes***

| What time last did you have a main meal (breakfast, lunch, or dinner)? |  |
| --- | --- |

***Menstruation***

| Are you currently menstruating? | *Yes No Not applicable* |
| --- | --- |
| How many days has it been since the end of your last menstrual period? | *[numerical] Don’t know Not applicable* |

***Mood and Anxiety (adapted from PHQ-4)***

| *Right now, are you feeling any of the following:* |  |  |  |  |
| --- | --- | --- | --- | --- |
| Feeling nervous, anxious or on edge | 0 (Not at all) | 1 (A little) | 2 (A lot) | 3 (Extremely) |
| Not being able to stop or control worrying | 0 (Not at all) | 1 (A little) | 2 (A lot) | 3 (Extremely) |
| Feeling down, depressed or hopeless | 0 (Not at all) | 1 (A little) | 2 (A lot) | 3 (Extremely) |
| Little interest or pleasure in doing things | 0 (Not at all) | 1 (A little) | 2 (A lot) | 3 (Extremely) |

***Mania (adapted from ASRM)***

| 1. Choose the statement that best describes your mood right now: | - I do not feel happier or more cheerful than usual. - I feel happier or more cheerful than usual. - I feel extremely happy or cheerful. |
| --- | --- |
| 1. Choose the statement that best describes your self-confidence right now: | - I do not feel more self-confident than usual. - I feel more self-confident than usual. - I feel extremely self-confident. |
| 1. Choose the statement that best describes your sleep patterns right now: | - I do not need less sleep than usual. - I need less sleep than usual. - I can go all day and night without any sleep and still not feel tired. |
| 1. Choose the statement that best describes your speech right now: | - I am not talking more than usual. - I am talking more than usual. - I talk constantly and cannot be interrupted. |
| 1. Choose the statement that best describes your activity level right now: | - I am not more active (either socially, sexually, at work, home or school) than usual. - I am more active than usual. - I am constantly active or on the go all the time. |

***Psychosis (adapted from BPRS^1^)***

| Is someone spying on you or plotting against you? | 0 (Not at all) | 1 (A little) | 2 (A lot) | 3 (Extremely) |
| --- | --- | --- | --- | --- |
| Can people read your thoughts, or can you read theirs? | 0 (Not at all) | 1 (A little) | 2 (A lot) | 3 (Extremely) |
| Do you feel possessed or is someone or something putting thoughts into your head? | 0 (Not at all) | 1 (A little) | 2 (A lot) | 3 (Extremely) |
| Are you getting special messages that are intended only for you? | 0 (Not at all) | 1 (A little) | 2 (A lot) | 3 (Extremely) |

***Sleep (Brief-PSQI)***

| How long did it take you to fall asleep last night? |  |
| --- | --- |
| How many hours of actual sleep did you get last night? |  |
| Have you had trouble sleeping because you wake up in the middle of the night or early morning? |  |
| How would you rate the quality of your sleep last night? | 0 (Very Good)  1 (Good) 2 (Bad) 3 (Very Bad) |

1 Moitra, E., Park, H. S., Ben-Zeev, D. & Gaudiano, B. A. Using ecological momentary assessment for patients with psychosis posthospitalization: Opportunities for mobilizing measurement-based care. *Psychiatric rehabilitation journal* **44**, 43 (2021).
